# Characteristics of Color Development in Seeds of Brown- and Yellow-Seeded Heading Chinese Cabbage and Molecular Analysis of *Brsc*, the Candidate Gene Controlling Seed Coat Color

**DOI:** 10.3389/fpls.2017.01410

**Published:** 2017-08-14

**Authors:** Yanjing Ren, Qiong He, Xiaomin Ma, Lugang Zhang

**Affiliations:** State Key Laboratory of Crop Stress Biology for Arid Area, College of Horticulture, Northwest A&F University Yangling, China

**Keywords:** Chinese cabbage, flavonoids, seed coat color, *BrTTG1*, sequence analysis, expression levels

## Abstract

The proanthocyanidin (PA) is the main flavonoids which affect the seed coat color in *Brassica* species. In this paper, characteristics of color development and accumulation of flavonoids were analyzed in the seeds of brown-seeded (B147) and yellow-seeded (B80) heading Chinese cabbage (*Brassica rapa* L. ssp. *Pekinensis*). It is found that the content of phenolic compounds in B147 were significantly more than that of B80 by using dimethylaminocinnamaldehyde (DMACA) staining and toluidine blue O (TBO) staining. In previous studies, the locus associated with seed coat color has been mapped. The results of whole genome re-sequencing showed that there are large fragment deletions variation in the mapping region between the brown-seeded parent ‘92S105’ and the yellow-seeded parent ‘91-125.’ Based on the *B. rapa* genome annotation information, the *TRANSPARENT TESTA GLABRA 1* (*TTG1*), is likely to be the candidate gene controlling seed coat color. A 94-base deletion was found in the 96th base downstream of the initiation codon in the *TTG1* of yellow seed, thus, the termination codon TGA was occurred in the 297th base which makes the full length of *TTG1* of yellow seed is 300 bp. Based on the differential sequences of *TTG1* of brown and yellow seed, a functional marker, Brsc-yettg1, was developed to detect the variation of *TTG1*. Quantitative real-time PCR analysis of *BrTTG1* in different tissues showed that expression levels of *BrTTG1* was not tissue-specific. During the whole seed development period, the expression of *BrTTG1* in B147 was higher than that of B80. The expression levels of four structural genes, *BrDFR, BrANS, BrANR1*, and *BrANR2* in B147 were also higher than those in B80. The co-segregation molecular markers obtained in this report and *TTG1* related information provide a basis for further understanding of the molecular mechanism of seed coat color in heading Chinese cabbage.

## Introduction

Yellow seed coat and brown seed coat are two kinds of seed coat color which were universally existed in Chinese cabbage (*Brassica rapa* L. ssp. *Pekinensis*). Yellow seed coat is typically a desirable quality trait in new hybrid breeding of *Brassica* species (Li et al., 2012; Wang et al., 2016), such as the higher quality of oil and meal (Stringam et al., 1974), lower fiber content and higher protein content (Shirzadegan and Röbbelen, 1985; Getinet et al., 1996). Proanthocyanidins (PAs), the oligomeric and polymeric end products synthesized via the flavonoid biosynthesis pathway (Winkel-Shirley, 2001; Dixon et al., 2005), are commonly found in the plant kingdom and generally have protective effects on predation and pathogen attack (Scalbert, 1991; Harborne and Grayer, 1993). Furthermore, their beneficial effects on human health have been demonstrated in several studies (Bagchi et al., 2000; Manach et al., 2005; Georgiev et al., 2014). In flavonoid biosynthetic pathway, genes (i.e., *CHS, CHI, F3H*, and *F3′H*) are involved in the production of common precursors (i.e., dihydroflavonols), which were named early biosynthetic genes (EBGs) (Xu et al., 2014). In addition, the downstream genes (i.e., *DFR, LDOX, ANR*) of the pathway are usually named late biosynthetic genes (LBGs) (Xu et al., 2015). Most of the corresponding genes have been detected to affect the formation of seed coat pigmentation, and may result in a transparent testa (tt) mutations (Lepiniec et al., 2006; Appelhagen et al., 2014; Ichino et al., 2014). In Brassicaceae, PAs were accumulated in the endothelium layer of the inner integument of seed coat, which caused the formation of brown seed coat color (Lepiniec et al., 2006).

Previous reports published more than one method for detecting PA and polyphenol compounds (Oszmianski and Bourzeix, 1996; Qu et al., 2013). Oszmianski and Bourzeix (1996) reported that dimethylaminocinnamaldehyde (DMACA) appeared most sensitive in the catechin and PA analysis than the Folin-Ciocalteau (FC) reagent and the vanillin. Hence, DMACA was used for detecting PAs in this paper. Spatiotemporal accumulation of polyphenol compounds in the seed coats of black- and yellow-seeded *Brassica napus* was measured by Qu et al. (2013) using toluidine blue O (TBO) staining of transverse sections of developing seeds, which results showed that polymeric phenolic compounds deposited mainly to the palisade and pigment layers in the seed coat of black-seeded. In contrast, these compounds were mainly localized near the hilum of the pigment layer in seed coats of yellow-seeded (Qu et al., 2013).

Many studies had reported that different genes were responsible for seed color coat in different *Brassica* species. In *B. rapa*, by mapping and blastn analysis with the *Arabidopsis* genome, Li et al. (2012) found that a large insertion in bHLH transcription factor *BrTT8* result in yellow seed coat. Wang et al. (2016) identified that *BrTT1* is a candidate gene for the seed coat color trait in variety of Dahuang via whole-genome re-sequencing. In *B. napus*, Fu et al. (2007) considered that the bifunctional gene *TT10* was an important candidate gene for seed coat color. Zhou et al. (2016) revealed that *BnaC.TT2* regulated the seed color formation. In *Brassica juncea*, Padmaja et al. (2014) reported that two homoeologous *TT8* genes control yellow seed coat trait in allotetraploid. Huang et al. (2016) identified the Bra036828, which is high similarity with the TRANSPARENT TESTA 6 gene, was possibly responsible for yellow seed color.

Different genotypes lead to the different materials and growth cycles for seed. Hence, in the present study, in order to further understand the diversity of seed coat color between two Chinese cabbage materials, we analyzed accumulation characteristic of PAs and flavonoids in different development period seeds. Furthermore, seed coat color gene was identified by whole-genome re-sequencing. We also analyzed the expression of seed coat color gene in different tissues and different development period seeds and the expression of genes related seed coat color formation.

## Materials and Methods

### Plant Materials

Two heading Chinese cabbage pure inbred lines, the brown-seeded line ‘92S105’ and the yellow-seeded line ‘91-125,’ provided by Chinese cabbage research group of Northwest A&F University, were crossed by artificial emasculation, then an F_2_ population was developed from self-breeding of a single heterozygous F_1_ individual plant. The F_2_ segregation population and two parental lines were planted in the experimental station of Northwest A&F University, Yangling, China, in 2013. The seed coat colors of individuals in the F_2_ population were divided into brown and yellow types by visual observation at the seed maturation stage. The F_3_ family populations derived from brown-seeded individuals of F_2_ by artificial self-pollination were planted in 2014, which were used to detect whether the genotypes of F_2_ individuals at seed color loci is homozygous or heterozygous. A self-compatible and homozygous F_3_ brown-seeded progeny B147 and yellow-seeded progeny B80 were selected and planted in 2015, which were used for collecting roots, shorted stems, rosette leaves, leaf petioles, flower stems, cauline leaves, flowers, buds, and different ripeness seeds of 8, 10, 12, 14, 16, 18, 20, 22, 24, 26, 28, 30, 32, 34 days after flowering (DAF). In addition, a new F_2_ population which derived from the hybridization of the other one yellow-seeded line and one brown-seeded line of heading Chinese cabbage was used for testing the accuracy of co-segregation marker, which number of individual is 270 progeny plants.

### Seed Coat DMACA Staining

Different ripeness period seeds of B147 and B80 at 8, 10, 12, 14, 16, 18, 20, 22, 24, 26, 28, 30, 32, 34 DAF were harvested and directly fixed in a FAA fixation solution (50% ethanol, 5% acetic acid, and 5% formaldehyde) for more than 24 h at 4°C. Then seeds were taken out from the fixation solution and were rinsed by distilled water. Seeds coat and embryo were separated by tweezers extrusion. Seeds coat were stained in 0.5% DMACA hydrochloric acid ethanol solution (0.5% DMACA, 15% hydrochloric acid, and 50% ethanol) for 3 h at room temperature and observed the changes of seed coat color.

### Tissue Section Observation

Referenced as Qu et al. (2013) report, 10 μm thick sections of B147 and B80 seeds at 10, 14, 28 DAF age were cut with a rotary microtome (LEICA, Germany), then stained with TBO and observed with a upright microscope (Olympus, Japan). Three replications of each material were sectioned.

### DNA/RNA Extraction

Total DNA of individuals were extracted from young fresh leaves by modified CTAB (cetyl trimethyl ammonium bromide) method (Porebski et al., 1997). The final DNA concentration was adjusted to 100 ng/μL with double distilled water. Total RNA was extracted from roots, shorted stems, rosette leaves, leaf petioles, flower stems, cauline leaves, flowers, buds, and different ripeness period seeds of brown-seeded line (B147) and yellow-seeded line (B80) with TaKaRa MiniBEST Plant RNA Extraction Kit (TaKaRa, Japan) according to the manufacturer’s instructions. The quality of RNA was assessed by electrophoresis with 0.8% agarose gel with staining in ethidium bromide solution. Genomic DNA elimination and the first-strand cDNA synthesis were performed with a PrimeScriptTM RT reagent Kit with gDNA Eraser (TaKaRa, Japan) following the manufacturer’s instructions and the final cDNA concentration was adjusted to 100 ng/μL with RNase free water.

### Whole Genome Re-sequencing

The genomic DNA was randomly broken into short DNA fragments with enzymes and repaired to a flunt end. Then dA tails and sequencing joints were connected at both ends of the DNA fragment one after another. DNA fragments with the joint were purified by AMPure XP beads and 300–400 bp length fragments were selected for PCR amplification. The library needed to be purified and tested, and sequenced by Hiseq X10 PE150 sequencing machine. Whole genome re-sequencing was performed by Guangzhou GENE DENOVO Biotechnology Co. Ltd (Guangzhou, China).

### Candidate Gene Prediction and Development of Functional Marker

The physical position of flanking markers linked with seed coat color were determined by the BLAST network online service and candidate gene prediction was performed using GBrowse tool from BRAD^1^. The specific primers for the candidate gene were designed on the basis of the reference genome of *B. rapa* (Wang et al., 2011). The sequences of the candidate gene were cloned from the ‘92S105’ and ‘91-125.’ The PCR products were purified using a TaKaRa MiniBEST agarose Gel DNA Extraction Kit (TaKaRa, Japan) and used for TA cloning. The purified PCR products were introduced into the pMD18-T Simple Vector (TaKaRa, Japan) and transformed into *Escherichia coli* strain DH5α. The recombinant plasmids were sequenced by Sunny Biotechnology, Co. Ltd (Shanghai, China) and sequence alignment was performed with DANMAN software. The complete genomic DNA with intronless region of genomic sequences were submitted to the GenBank under the following accession numbers: 92S105 (KY929015) and 91-125 (KY929016). The function of the predicted gene was searched by the BLASTP tool from NCBI^2^.

Based on the variation in sequences of *TTG1* of brown seed (*TTG1-B*) and *TTG1* of yellow seed (*TTG1-Y)*, a specific pair primer, was designed and synthesized, which amplified a functional marker Brsc-yettg1. The PCR amplifications were performed for detecting the *TTG1* variation in two parents and other yellow-seeded progeny plants.

### Gene Expression Analysis

The analysis of genes expression were performed with quantitative real-time PCR (qPCR). A house-keeping gene encoding glyceraldehyde-3-phosphate dehydrogenase (GAPDH, GO0048316) was used as the reference gene (Li et al., 2016). The qPCR reactions was performed with 20 μL volume reaction solution included 2 μL of cDNA template (100 ng/μL), 0.5 μL of each primers (10 μmol), 10 μL 2× RealStar Green Power mixture (TaKaRa, Japan), and 7 μL double distilled water in an iQTM5 Multicolor Real-Time PCR Detection System (Bio-Rad, United States). The relative expression levels of genes were measured by 2^-ΔΔC_t_^ method (Livak and Schmittgen, 2001). The cDNA sequences of qPCR primers are listed in **Table 1**.

**Table 1:** All primers sequences for quantitative real-time PCR.

Primers name	Forwarding sequences 5′–3′	Reversed sequences 5′–3′
GAPDH	TAACTGCCTTGCTCCACTTGC	CGGTGCTGCTGGGAATGAT
BrTTG1	TGTATGGCGACGATTCTGA	CAATCCCATTCGGTCCAG
BrDFR	GCTACGATGACGCCATAAAC	TTCCAGCAGACGAAGTAAACAC
BrANR1	ATCAATCCAGCGATACAAGG	TTCGGTCATCACAAGTCCAG
BrANR2	GTGCTTATCAAGTGCGAAAC	CTCCGTCATCTCATCATACA
BrANS	TATTACCCGAAATGCCCTCAG	ACAGCCCAAGAAATCCTTACCT

## Results

### Seed Coat Color Changes in Seed Ripening Process and DMACA Staining

**FIGURE 1:**
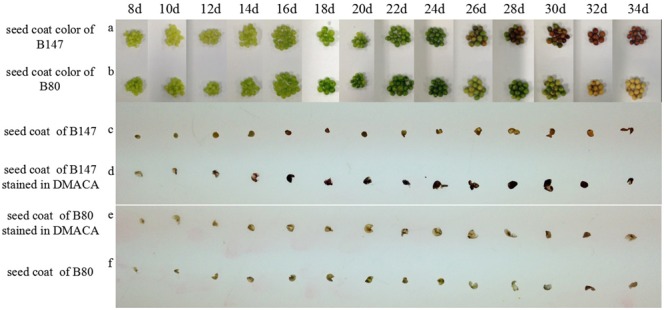
Seed coat color changes during seed development and DMACA staining. **(a,b)** Seed coat color change during seed development in brown-seeded line B147 and yellow-seeded line B80 *Brassica rapa*. **(c,f)** Seed coat color in B147 and B80 during seed ripening period fixed by FAA. **(d,e)** Seed coat color changes in B147 and B80 after staining in DMACA solution during seed ripening period.

Different ripeness period seeds of B147 and B80 were selected to observe and take pictures (**Figure 1**). The seed coat color of B147 (**Figure 1a**) was pale green from 8 to 14 DAF, then became green at 16 DAF and emerged brown color at 26 DAF, finally became full brown at 32 DAF. The seed coat color of B80 (**Figure 1b**) was also pale green from 8 to 14 DAF and becoming green at 16 DAF, but emerging yellow color at 26 DAF, finally became full yellow color at 32 DAF.

Seed coats of B147 and B80 stained with DMACA were shown in **Figures 1d,e**. PAs in the seed coat specifically reacted with DMACA and produced blue products, therefore the shades of blue color may reflect PAs content in corresponding tissue. Seeds coat color of B147 stained with DMACA (**Figure 1d**) had no difference with control (**Figure 1c**) at 8 DAF to 12 DAF, but became light blue at 14 DAF, then became deep blue from 16 DAF to ripe seed. After DMACA staining, the seed coat color began to change at 14 DAF, which was earlier than the color change of ripe seed in natural at 26 DAF. This result indicated that PAs in seed coats of B147 has produced at 14 DAF, but its phenotype color can be investigated at 26 DAF. Compared with B147, seeds coat color of B80 stained with DMACA (**Figure 1e**) had no difference with control (**Figure 1f**) in all seed ripening stage from 8 to 34 DAF, which suggested that PAs was little or not produced in seed coats of B80 during seed ripening.

### Accumulation of Flavonoids in the Seed Coat

**FIGURE 2:**
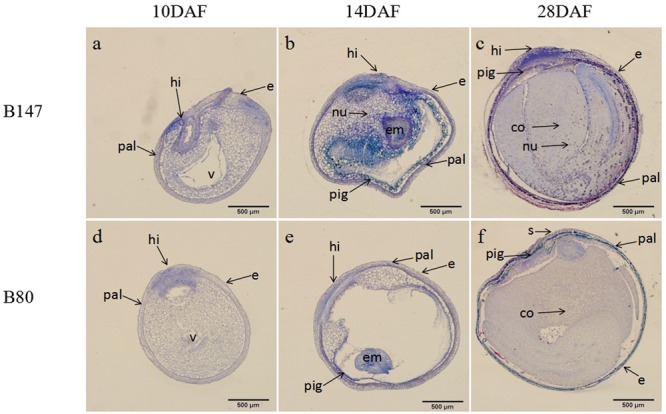
Changes of flavonoids during seed development of brown-seeded and yellow-seeded *B. rapa*. **(a–c)** Distribution of flavonoids in seed coat development at 10 DAF, 14 DAF, and 28 DAF in B147. **(d–f)** Distribution of flavonoids in seed coat development at 10 DAF, 14 DAF, and 28 DAF in B80. Black arrows, accumulation site for flavonoids; pal, palisade layer; hi, hilum; e, epidermis; nu, nucleus; v, vacuole; em, embryo; pig, pigment layer; s, subepidermis; co, cotyledon.

According to the color changes of B147 seed coat stained with DMACA above, seeds of B147 and B80 at three stage of 10 DAF, 14 DAF, 28 DAF were selected for histochemical investigation. TBO staining transverse sections of seeds revealed that flavonoids began to deposit in the hilum of both seeds at 10 DAF (**Figures 2a,d**). The blue color of B147 in hilum stained was deeper than B80, indicating that more accumulation of flavonoids was in B147. At 14 DAF, the blue color of seeds stained extended to palisade layer, pigment layer and embryo (**Figures 2b,e**) and color of palisade layer and pigment layer in B147 seed stained more intensely than B80, suggesting that more flavonoids had deposited in B147 than B80. At 28 DAF, the color of palisade layer and pigment layer in B147 seed stained deeper (**Figure 2c**), but little change in B80 seed (**Figure 2f**), which indicated that the accumulation of flavonoids had raised more significantly in the palisade and pigment layers of B147 seeds than in those of B80.

In general, **Figure 2** showed that flavonoids gradually increased during seed maturation and showed significant differences in the seed coat of B147 and B80. The flavonoids deposited only in hilum in the seed of B147 and B80 at 10 DAF, then extended to the palisade layer and pigment layers at 14 DAF. The significant differences of flavonoids content in the seed coat between B147 and B80 occurred at 14 DAF, which suggested that more flavonoids deposited in B147 seed from this stage, but little change in B80 seed. These results were in agreement with the seed coat color change of B147 and B80 stained with DMACA.

### Seed Coat Color Candidate Gene Identification by Whole Genome Re-sequencing

**FIGURE 3:**
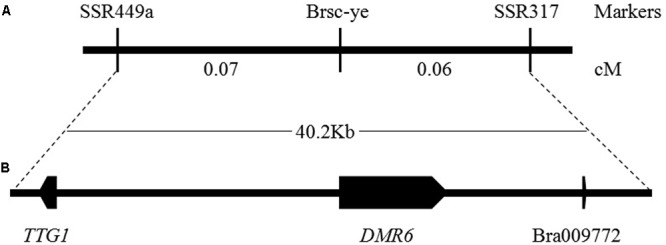
Genetic and physical maps of the *Brsc-ye* gene and the candidate gene identification. **(A)** Fine mapping of the *Brsc-ye* gene. The *Brsc-ye* gene was mapped to the region between markers SSR449a and SSR317 (Ren et al., 2017). **(B)** Candidate region of the *Brsc-ye* locus. The *Brsc-ye* locus was delimited to a 40.2 kb region in the *B. rapa* scaffold and there were three predicted genes in this region based on the genome sequence of *B. rapa.*

The genome sequences of the parental line, the brown-seeded line ‘92S105’ and the yellow-seeded line ‘91-125,’ were obtained by whole genome re-sequencing (the statistics of whole genome re-sequencing were shown in **Supplementary Table S1**). Based on the previous study, the locus linked with seed coat color had been mapped to a region of 40.2 Kb in A06 chromosome (Ren et al., 2017). Thus, comparing with the *B. rapa* variety chiifu genome sequence (Wang et al., 2011), it was found that there are three large fragment deletion in this range of the 40.2 Kb region (**Figure 3A**) of ‘92S105,’ therefore, the actual physical distance between the marker SSR449a and SSR317 shorten to 26.2 Kb. Similarly, there are two large fragment deletion in this range of ‘91-125,’ and the actual physical distance between the marker SSR449a and SSR317 also shorten to 23 Kb. Based on whole genome sequence of ‘92S105,’ ‘91-125’ and Brassica database^1^, there are three predicted genes in this corresponding chromosome region, *TRANSPARENT TESTA GLABRA 1* (*TTG1*), *DOWNY MILDEW RESISTANT 6* (*DMR6*) and a unknown protein (Bra009772) (**Figure 3B**). *TTG1* is a transcription factor that belongs to the WD40-repeat family protein and is involved in formation of MBW complexes. Thus, *TTG1* was considered as the candidate gene for seed coat color.

### Cloning and Analysis of Candidate Gene Sequence

Compare the *TTG1* genome sequence between ‘92S105’ and ‘91-125,’ then sequences of *TTG1* were cloned, sequenced and named as *TTG1-B* and *TTG1-Y* for ‘92S105’ and ‘91-125,’ respectively. A 94-base deletion was found in the 96th base downstream of the initiation codon in the *TTG1-Y*, which caused the termination codon to appear at 297th base. Thus, the full length of *TTG1* of yellow seed is 300 bp (sequence alignment result was showed in **Supplementary Figure S1**). Amino acid sequence prediction indicated that the full protein of 337 amino acids were encoded by *TTG1-B*, while a curtate protein of 99 amino acids was encoded by *TTG1-Y* (**Supplementary Figure S2**). The full protein of *TTG1* includes four WD40 structures, which are located in the position of amino acids residues from 60th to 105th, 112th to 157th, 160th to 198th, 249th to 289th (**Supplementary Figure S3**), respectively. However, no WD40 structure was found in the curtate protein coded by *TTG1-Y*.

### Functional Marker of Seed Coat Color

Based on the sequence difference between the *TTG1-B* and the *TTG1-Y*, a new pair of SSR primer, named as Brsc-yettg1, was designed and was used for testing the consistency between seed coat color phenotypes and product of Brsc-yettg1 in 270 F_2_ individuals, which showed that the polymorphic bands of Brsc-yettg1 was completely consistent with the seed coat color, as a co-segregated marker (**Supplementary Figure S4**). This result further indicated that *TTG1* is the candidate gene controlling yellow-seeded trait. The co-segregated markers, Brsc-yettg1, will be useful in MAS breeding of yellow-seeded Chinese cabbage.

### Expression of *BrTTG1* Gene

**FIGURE 4:**
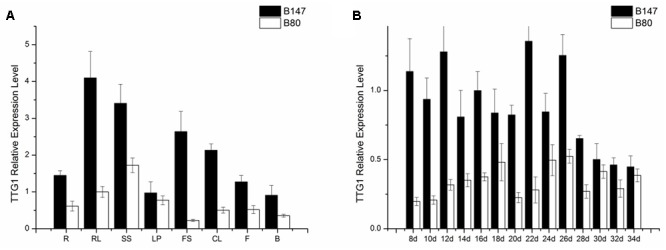
Expression level of *BrTTG1* in brown-seeded line B147 and yellow-seeded line B80. **(A)** Expression level of *BrTTG1* in different tissues (R, root; SS, shorted stem; RL, rosette leaf; LP, leave petiole; FS, flower stem; CL, cauline leaf; F, flower; and B, bud). **(B)** Expression level of *BrTTG1* in seed of B147 and B80 at different ripening period (8, 10, 12, 14, 16, 18, 20, 22, 24, 26, 28, 30, 32, and 34 DAF).

*TTG1* is thought to be the candidate gene controlling seed coat color, therefore, the *TTG1* transcript level in different tissues (roots, shorted stems, rosette leaves, leaf petioles, flower stems, cauline leaves, flowers, buds) of B147 and B80 were analyzed by qPCR. Results showed that *BrTTG1* expressed in all organs tested and had higher expression in B147 than in B80. In tissues of B147, the highest expression was in rosette leaf and the lowest expression was in bud (**Figure 4A**). The order of *BrTTG1* expression level from high to low is rosette leaf > shorted stem > flower stem > cauline leaf > root > flower > leave petiole > bud. In tissues of B80, the highest expression was in shorted stem and the lowest expression was in flower stem (**Figure 4A**). The order of *BrTTG1* expression level from high to low is shorted stem > rosette leaf > leave petiole > root > flower > cauline leaf > bud > flower stem in B80.

Further, *BrTTG1* expression patterns in different ripeness seeds at 8, 10, 12, 14, 16, 18, 20, 22, 24, 26, 28, 30, 32, and 34 DAF of B147 and B80 were performed by qPCR. Results showed that the expression level of *BrTTG1* in B147 was higher than that in B80 in the all developing stages. In the process of seed growth, the tendency of *BrTTG1* expression appeared a wavelike in both of B147 and B80, which has no relationship with the changes of seed color in them. Among them, the expression of *BrTTG1* in B147 was five times higher than that in B80 at 8 DAF. When the seeds grow to 30 DAF, there was no difference in the expression of *BrTTG1* between B147 and B80 (**Figure 4B**).

### Expression of Genes Involved in Flavonoids Biosynthesis

**FIGURE 5:**
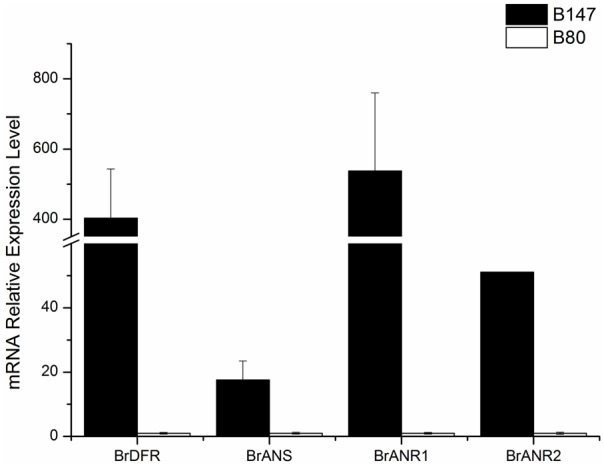
Expression levels of four late biosynthetic genes (LBGs), *BrDFR, BrANS, BrANR1*, and *BrANR2* in seeds of brown-seeded line B147 and yellow-seeded line B80.

Four LBGs (*BrDFR, BrANS, BrANR1*, and *BrANR2*) involved in flavonoids biosynthesis were measured in seeds at 30 DAF of B147 and B80. The relative transcription levels were showed in **Figure 5** referenced by B80. The expression of *BrDFR* in B147 was approximate 400-fold of that in B80, and the expression of *BrANS* in B147 also increase 17-fold of that in B80. The expression of *BrANR1* and *BrANR2* in B147 were nearly 500-fold and 50-fold higher than those in B80, respectively. All these results indicated that the *TTG1-Y* mutation seriously debased the transcript level of LBGs in flavonoids pathway, and maybe results in the formation of yellow seed coat.

## Discussion

In Brassicaceae, Lepiniec et al. (2006) reported that the formation of brown seed coat was due to the accumulation of PAs in the endothelium layer of the inner integument. In *B. napus*, the seed coat color is affected by the content of phenolic compounds cyanidin and procyanidins (Marles and Gruber, 2004; Akhov et al., 2009; Nesi et al., 2009). By the TBO staining of seed coat color, phenolic compounds mainly localized to hilum, palisade layer, epidermis and pigment layer in brown seed, while these compounds were mainly distributed to hilum in yellow seed, which is consistent with the reports of Qu et al. (2013). However, the period of accumulation of PAs and phenolic compounds in this paper showed differences between with the reports of Qu et al. (2013). They reported that phenolic compounds began to accumulate in the hilum at 14 DAP and in the palisade layer at 21 DAF in brown seed coat in *B. napus*, but these compounds began to deposit in the hilum of seeds at 10 DAF and in the palisade layer and pigment layer at 14 DAF in *B. rapa* in this paper. Furthermore, the time of seed maturation also showed different between them. The time of seed maturation of *B. napus* in report of Qu et al. (2013) is more than 49 DAP, while the time of *B. rapa* is just 34 DAF in this paper.

Many reports had suggested that different yellow seed sources may own special gene for seed coat color. In *B. napus*, the bifunctional gene *TT10* (*TRANSPARENT TESTA10*) (Fu et al., 2007) and *BnaC.TT2* (Zhou et al., 2016) were reported to be important candidate genes for seed coat color. In *B. juncea*, two homoeologous *TT8* genes (Padmaja et al., 2014) and the Bra036828 (Huang et al., 2016) were identified for yellow seed color. In *B. rapa*, the transcription factor *BrTT*8 (Li et al., 2012) and *BrTT1* (Wang et al., 2016) were thought to be possibly responsible for the seed coat color. All above genes reported for seed coat color are different. Of them, only one gene, Bra036828, which is high similarity with the *TRANSPARENT TESTA 6* gene, may be a flavonoid biosynthetic gene, others are regulatory genes, such as *TT2* (MYB123), *TT8* (bHLH042), *TT1* (WIP1/Zn finger) (Xu et al., 2015), which suggest that the formation of seed coat color is mainly controlled by regulatory genes rather than structural genes. MYB-bHLH-WDR complexes was formed by interactions of these transcription factors, whose function in flavonoid biosynthetic pathway is regulating the flavonoid biosynthetic gene and affecting formation of seed coat color (Xu et al., 2015).

*TTG1*, as a WD40 repeat transcription factor, plays multiple roles in different metabolism pathway, mainly controlling trichome initiation (Walker et al., 1999), seed coat mucilage production (Western et al., 2001), seed oil and storage protein accumulation (Chen et al., 2015), and plant growth and development (Liu et al., 2017). Qu et al. (2013) reported that the highest level of *TTG1* expression in 35 DAP seed of *B. napus*. However, the highest level of *TTG1* expression is in 22 DAF seed in this paper. The expression of genes involving in flavonoid biosynthetic pathway were also measured (Liu et al., 2013; Qu et al., 2013). Liu et al. (2013) conducted the expression levels of these genes in *B. juncea* by transcriptome analysis, which showed that *BjDFR, BjANS*, and *BjANR* genes were not expressed at all or at a very low level in the yellow-seeded testa. Qu et al. (2013) revealed that *BnDFR* and *BnANS* were expressed in brown and yellow seed and the expressions of them in brown seed were higher than that in yellow seed. In this study, qPCR was also used for detecting the expression level of LBGs, the similar results as reports of Liu et al. (2013) was appeared. The expression levels of *BrDFR, BrANS, BrANR1*, and *BrANR2* in brown seed were higher than these in yellow seed and these genes were at a very low expression level in yellow seed, which indicated that mutation of *TTG1* directly affect the expression levels of LBGs and influence formation of seed coat color. Although the regulation of MBW complexes for seed coat color has been discovered in many species, such as *Arabidopsis* and *Medicago truncatula* (Appelhagen et al., 2011a,b; Liu et al., 2014). The regulatory mechanism between these structural genes and transcription factors in *Brassica* species was hitherto unknown. Additional studies on understanding of the interactions of genes involved in seed coat color formation of *B. rapa* should be continued. The information obtained in this study will lay a foundation for understanding the molecular mechanism of yellow seed formation and MAS of yellow-seeded line breeding in *B. rapa*.

## Author Contributions

LZ and YR conceived the experiments; YR performed the experiments; QH participated in the analysis of genes expression; XM participated in collecting seeds; LZ contributed materials, reagents, and analysis tools; YR and LZ wrote the paper.

## Conflict of Interest Statement

The authors declare that the research was conducted in the absence of any commercial or financial relationships that could be construed as a potential conflict of interest.

## Footnotes

1 http://brassicadb.org/brad/

2 http://blast.ncbi.nlm.nih.gov/Blast.cgi

## Supplementary Material

FIGURE S1 Sequence alignment of *TTG1* in the brown- seeded parent 92S105 (*TTG1-B*) and in the yellow-seeded parent 91-125 (*TTG1-Y*). The accession numbers of *TTG1* in 92S105 and 91-125 were KY929015 and KY929016, respectively.

FIGURE S2 Predicted amino acid difference sequence encoded by *TTG1-B* and *TTG1-Y*.

FIGURE S3 Diagrammatic sketch of four WD40 structures of the TTG1 protein in the brown-seeded 92S105.

FIGURE S4 Polymorphism of co-segregation marker Brsc-yettg1 in the brown-seeded parent 92S105, yellow-seeded parent 91-125 and F_2_ individuals. B: brown-seeded parent 92S105, Y: yellow-seeded parent 91-125, F_2_: F_2_ individuals.

TABLE S1 The summary of whole genome re-sequencing.
