# Supplementary figures and images for: Characteristics of Color Development in Seeds of Brown- and Yellow-Seeded Heading Chinese Cabbage and Molecular Analysis of Brsc, the Candidate Gene Controlling Seed Coat Color
Source: Front Plant Sci. 2017 Aug 14;8:1410. doi: 10.3389/fpls.2017.01410 (PMC5558542; doi:10.3389/fpls.2017.01410)

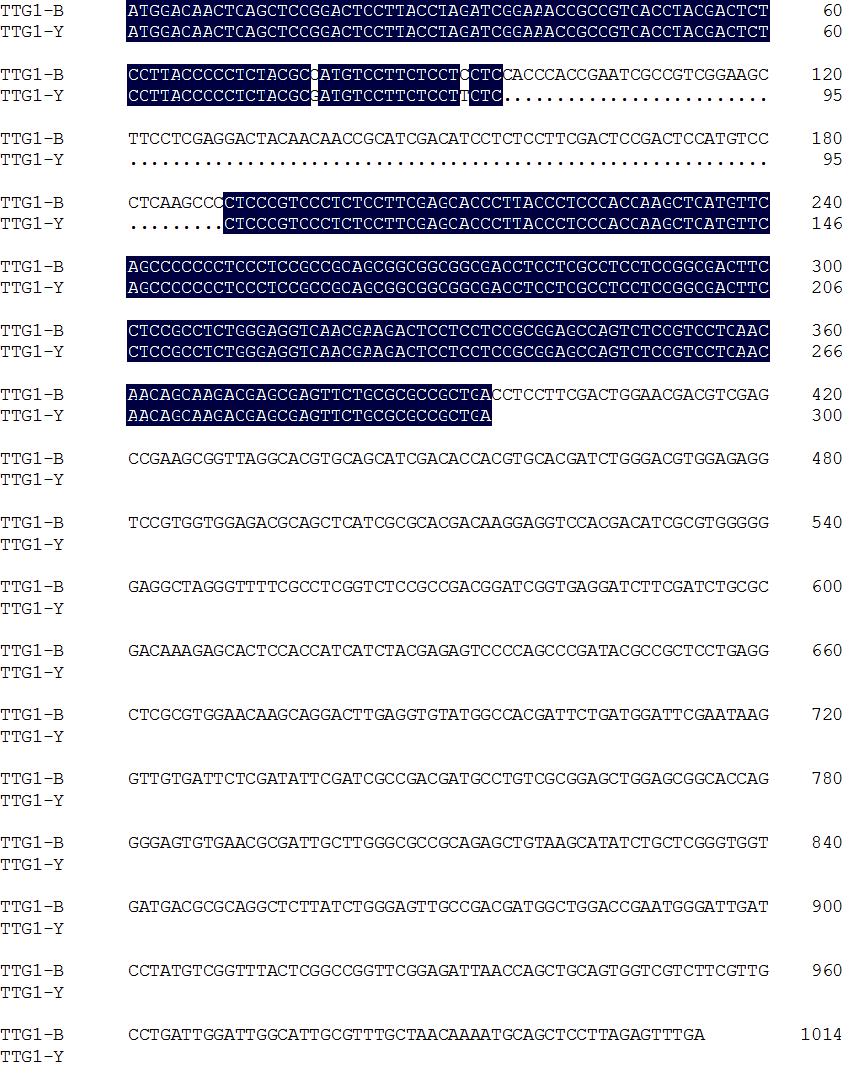

Supplement: FIGURE S1 — Sequence alignment of TTG1 in the brown- seeded parent 92S105 (TTG1-B) and in the yellow-seeded parent 91-125 (TTG1-Y). The accession numbers of TTG1 in 92S105 and 91-125 were KY929015 and KY929016, respectively. [file Image_1.JPEG]

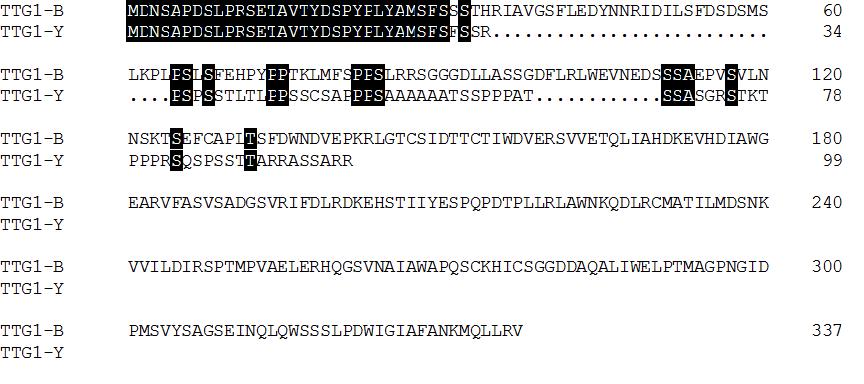

Supplement: FIGURE S2 — Predicted amino acid difference sequence encoded by TTG1-B and TTG1-Y. [file Image_2.JPEG]

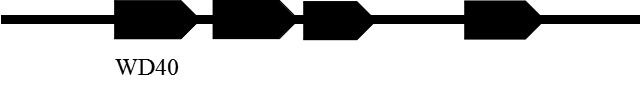

Supplement: FIGURE S3 — Diagrammatic sketch of four WD40 structures of the TTG1 protein in the brown-seeded 92S105. [file Image_3.JPEG]

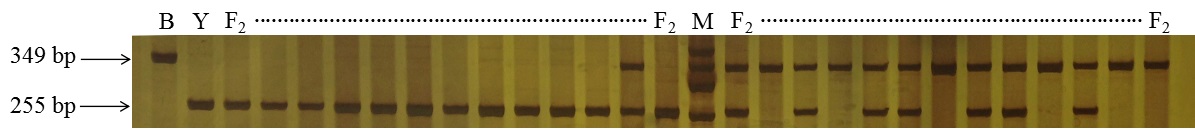

Supplement: FIGURE S4 — Polymorphism of co-segregation marker Brsc-yettg1 in the brown-seeded parent 92S105, yellow-seeded parent 91-125 and F2 individuals. B: brown-seeded parent 92S105, Y: yellow-seeded parent 91-125, F2: F2 individuals. [file Image_4.JPEG]
