# Supplementary material for: Characteristics of Color Development in Seeds of Brown- and Yellow-Seeded Heading Chinese Cabbage and Molecular Analysis of Brsc, the Candidate Gene Controlling Seed Coat Color
Source: Front Plant Sci. 2017 Aug 14;8:1410. doi: 10.3389/fpls.2017.01410 (PMC5558542; doi:10.3389/fpls.2017.01410)
Supplement: TABLE S1 — The summary of whole genome re-sequencing. [file Table_1.DOCX]

**Characteristics of Color Development in seeds of Brown- and Yellow-seeded Heading Chinese Cabbage and Molecular Analysis of *Brsc*, the Candidate Gene Controlling Seed Coat Color**

Yanjing Ren, Qiong He, Xiaomin Ma, Lugang Zhang*

State Key Laboratory of Crop Stress Biology for Arid Area, College of Horticulture, Northwest A&F University, Yangling, Shaanxi, PR China

*Corresponding author: Lugang Zhang

E-mail: [lugangzh@163.com](mailto:lugangzh@163.com;)

[Tel.: +86](Tel:086)-029-87082131; Fax: +[86](Tel:086)-029-87082163

Table S1：The summary of whole genome re-sequencing

| Plant materials | Clean Data(bp) | Clean Reads Number | Align Ratio(%) |
| --- | --- | --- | --- |
| 92S105 | 62360349648 | 412982448 | 82.86 |
| 91-125 | 11665962000 | 77773080 | 90.48 |
